# Intrinsically Photosensitive Retinal Ganglion Cells of the Human Retina

**DOI:** 10.3389/fneur.2021.636330

**Published:** 2021-03-25

**Authors:** Ludovic S. Mure

**Affiliations:** ^1^Institute of Physiology, University of Bern, Bern, Switzerland; ^2^Department of Neurology, Zentrum für Experimentelle Neurologie, Inselspital University Hospital Bern, Bern, Switzerland

**Keywords:** retina, retinal ganglion cell, intrinsically photosensitive ganglion cell, melanopsin (OPN4), non-visual responses to light

## Abstract

Light profoundly affects our mental and physical health. In particular, light, when not delivered at the appropriate time, may have detrimental effects. In mammals, light is perceived not only by rods and cones but also by a subset of retinal ganglion cells that express the photopigment melanopsin that renders them intrinsically photosensitive (ipRGCs). ipRGCs participate in contrast detection and play critical roles in non-image-forming vision, a set of light responses that include circadian entrainment, pupillary light reflex (PLR), and the modulation of sleep/alertness, and mood. ipRGCs are also found in the human retina, and their response to light has been characterized indirectly through the suppression of nocturnal melatonin and PLR. However, until recently, human ipRGCs had rarely been investigated directly. This gap is progressively being filled as, over the last years, an increasing number of studies provided descriptions of their morphology, responses to light, and gene expression. Here, I review the progress in our knowledge of human ipRGCs, in particular, the different morphological and functional subtypes described so far and how they match the murine subtypes. I also highlight questions that remain to be addressed. Investigating ipRGCs is critical as these few cells play a major role in our well-being. Additionally, as ipRGCs display increased vulnerability or resilience to certain disorders compared to conventional RGCs, a deeper knowledge of their function could help identify therapeutic approaches or develop diagnostic tools. Overall, a better understanding of how light is perceived by the human eye will help deliver precise light usage recommendations and implement light-based therapeutic interventions to improve cognitive performance, mood, and life quality.

## Introduction

The last years have seen an increased awareness of the impact of light on health, particularly of its detrimental effects when light is not delivered at the appropriate time. Light at night, also called “light pollution,” is becoming a major environmental and health concern (1–4). Even low-level light exposure from light-emitting devices, smartphones, or tablets may disrupt sleep (5, 6). As inappropriate illumination can be detrimental to health, optimal lighting can be a simple, cost-efficient population-level intervention to improve health: if light is delivered at the right time and in the right amount, it can ameliorate the quality of life in the nursing home and improve cognitive performances at school and at work (7–9).

Both beneficial and detrimental effects of light are mediated not only by rods and cones, the well-known photoreceptors that serve vision but also by a third class of cells in our retina. These cells are a subset of retinal ganglion cells (RGCs) expressing the photopigment melanopsin that renders them sensitive to light. They have been referred to as either photosensitive, intrinsically photosensitive retinal ganglion cells (pRGCs, ipRGCs), or melanopsin-expressing retinal ganglion cells (mRGCs) according to the context, i.e., when the studies focus on their response to light or on the presence of melanopsin respectively. Here, for simplicity, I will use the acronym ipRGCs. ipRGCs play a major role in what is called “non-visual” or “non-image-forming” responses to light. These responses include the alignment of our internal clock to the environmental day/night cycle, the regulation of the sleep-wake cycles, of the pupillary reflex to light (PLR), and the modulation of mood (10–12). More recently, it has been shown that melanopsin-driven response of ipRGCs also participates in some aspects of vision (13–16).

Twenty years after their discovery (17, 18), ipRGCs are well-documented in rodents and have been reviewed in depth elsewhere (19–21). Although there are only a few thousand ipRGCs per retina, they exhibit remarkable heterogeneity. They differ regarding dendritic arborization, expression levels of melanopsin, brain targets, and light response properties. In the mouse retina, six different morphological subtypes (M1 through M6) have been characterized and at least five functional subtypes are described. While the M1 subtype expresses high levels of melanopsin, the M2–M6 subtypes express lower amounts of melanopsin and also exhibit reduced intrinsic photosensitivity. Accordingly, each ipRGC subtype is thought to execute distinct light-regulated functions at specific levels of light intensity or time constants. For example, a fraction of M1 ipRGCs mediates the photoentrainment of the circadian clock while M4 ipRGCs are involved in the effect of light on mood. In contrast, all ipRGC subtypes seem to project to visual structures [dLGN, superior colliculus (SC)], and it is believed that they all participate in some aspects of vision. Finally, while ipRGCs are the principal conduits for all light input to the non-image-forming visual responses, anatomical and electrophysiological evidence suggests that ipRGCs also receive input from rod/cone photoreceptors.

**Figure 1:**
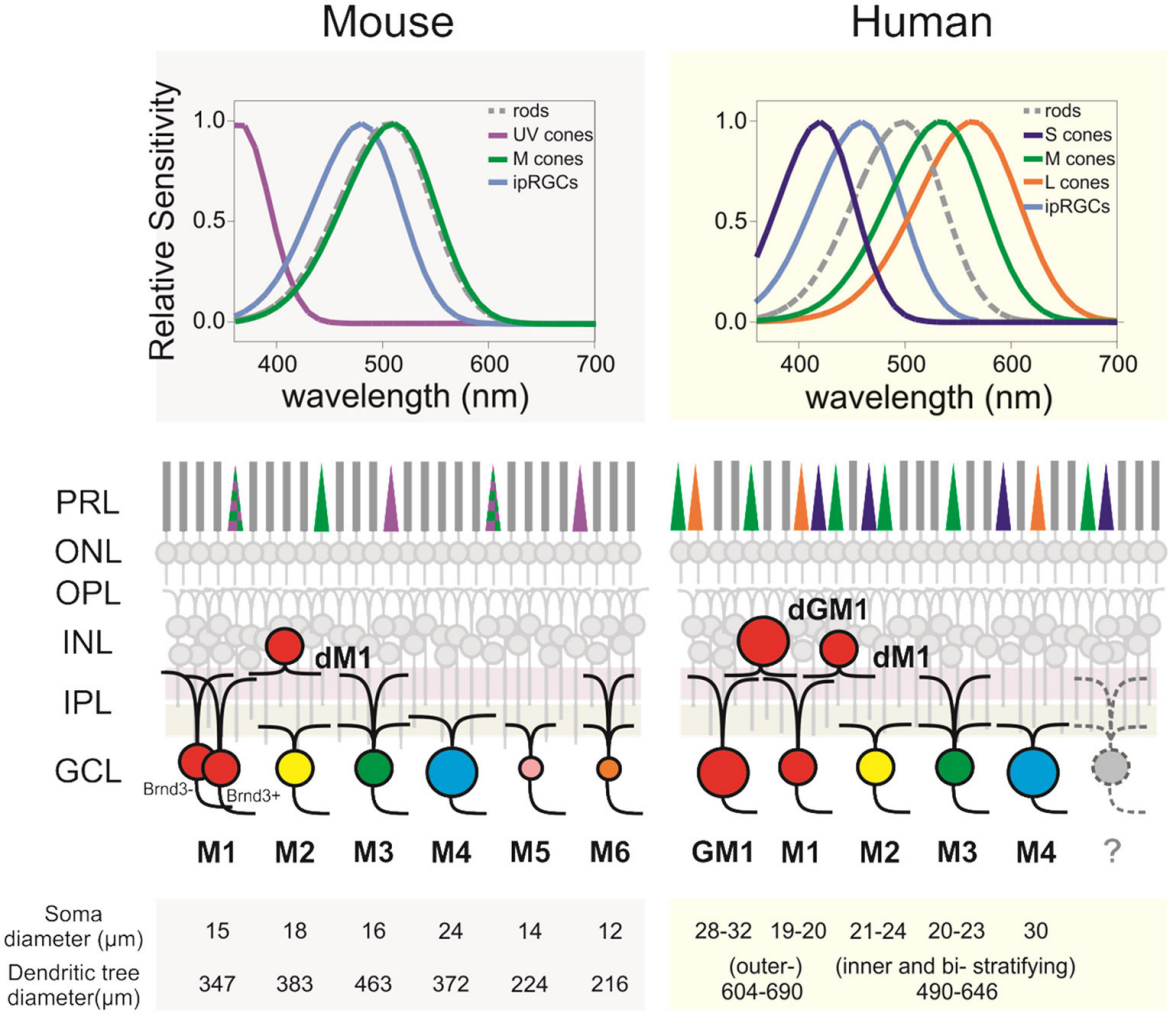
ipRGCs in the mouse and human retinas. (Upper panels) Relative spectral sensitivity of the rods, cones, and ipRGCs. (Middle panels) Diagram of murine and human retinas displaying the differences regarding the morphological subtypes of ipRGCs, their IPL dendritic stratification, and outer retina photoreceptors. (Lower panels) Morphological comparison between subtypes and species. Soma and dendritic tree measurements are rounded to the closest integer. Mouse data are from Sondereker et al. (21) that compiled them from literature. Human data are from Esquiva et al. (22), Hannibal et al. (23), Liao et al. (24), and Nasir-Ahmad et al. (25). GM1, gigantic M1; dM1, displaced M1; dGM1, displaced gigantic M1; PRL, photoreceptors layer; ONL, outer nuclear layer; OPL, outer plexiform layer; INL, inner plexiform layer; IPL, inner plexiform layer; GCL, ganglion cells layer.

In stark contrast to rodent ipRGCs, the exploration of ipRGCs in primates and in human, in particular, was, until recently, extremely limited. There is, however, a strong rationale to study them. Human and mouse are respectively diurnal and nocturnal animals. Human retina differs from the rodent retina on several levels, from the regional specialization of the retina to photoreceptor types and distribution (Figure 1). Human retina is adapted for high definition, color vision. This is achieved thanks to the fovea, a central zone of the retina (~1.2 mm of diameter), where three types of cones are densely packed. These cones (S, M, and L for short-, middle-, and long-wavelength cones) mostly express a unique photopigment with absorption peaks at 430, 531, and 561 nm, respectively (26, 27). In contrast, laboratory mice are nocturnal and their retina, devoid of fovea, is largely dominated by rods and expresses only two types of cone opsins [S- and M-opsin, with peak sensitivities at 360 nm and 508 nm, respectively (28, 29) often co-expressed in the same cone (30). As a consequence, there is a lack of appropriate murine models for some humane ocular disorders, such as age-related macular degeneration (31). Apart from anatomical discrepancies, there is also the genetic gap between the two species, which may result in different phenotypes in some cases of genetically inherited diseases (32). Another caveat is human modern lifestyle that results in a number of disorders such as diabetic retinopathy, which does not naturally occur in rodents.

Fortunately, the gap of knowledge in human ipRGCs is progressively being filled. New approaches and techniques have allowed characterizing morphological and functional human ipRGC subtypes, their transcriptome, and realizing that, in several disorders, they are either more resilient or vulnerable than conventional RGCs. The present paper reviews this recent progress in our knowledge of human ipRGCs, briefly compares their characteristics with those of the most studied model, the laboratory mouse, and highlights some outstanding questions and future challenges.

## Human ipRGCs Comprise Several Morphological Subtypes

Shortly after its discovery in the mouse, melanopsin was also found in the human inner retina (33). Melanopsin expression was detected in a subpopulation of RGCs located in the ganglion cell layer but also sometimes displaced in the inner nuclear cell layer. Melanopsin-expressing cells have a particular morphology with two to four dendritic processes constituting an extensive network throughout the retina. Melanopsin immunoreactivity is present in the soma and neuronal processes membranes and, to some extent, in the cytoplasm (33–35). Rare melanopsin-positive cones were also described in the human retina (36).

**Figure 2:**
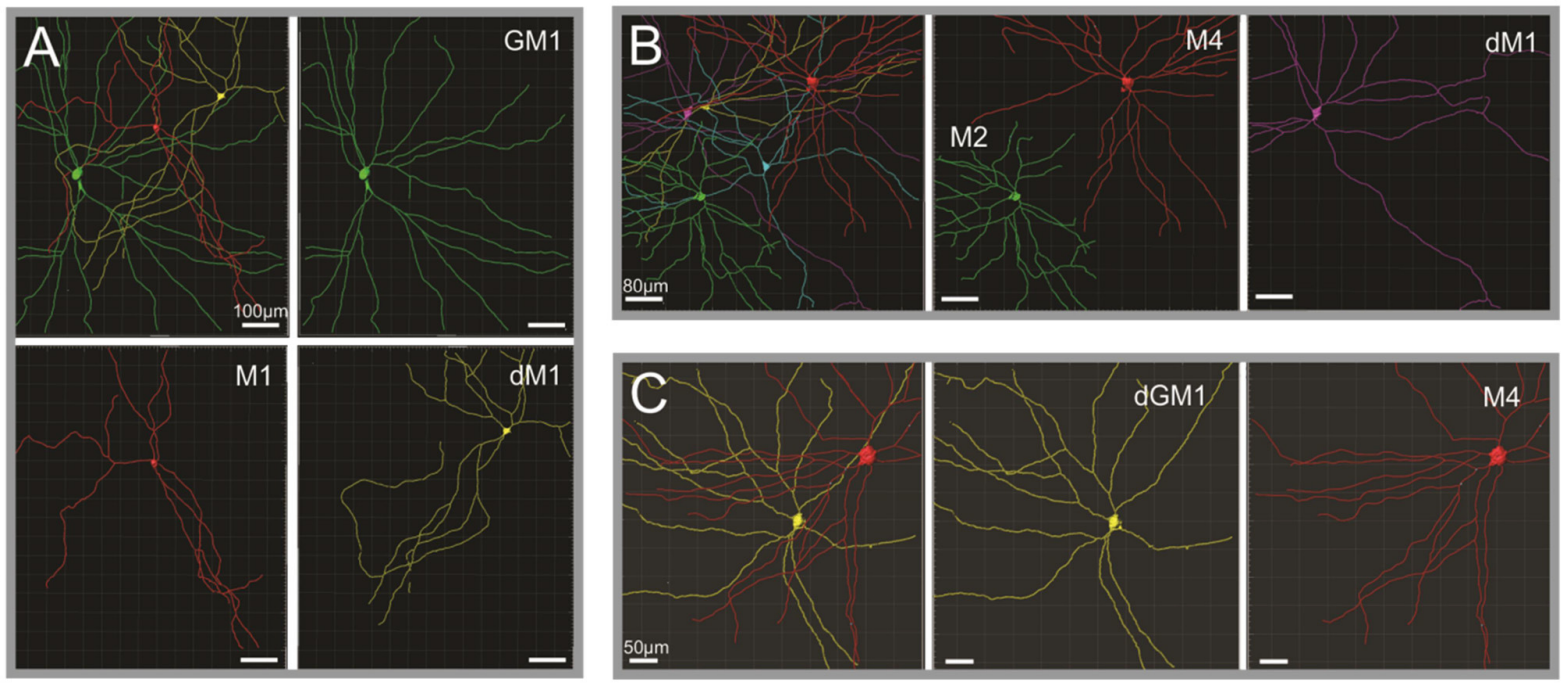
Human ipRGCs morphological subtypes. **(A–C)** Reconstruction and pseudocoloring of ipRGCs from three separate human retina volumes based on melanopsin immunoreactivity. Upper left subpanels illustrate the different ipRGCs detected in the volumes, their relative size, and arrangement toward each other. In the other subpanels, each ipRGC is then identified and represented separately to appreciate the details of their dendritic arborization. dM1, displaced M1; GM1, gigantic M1; dGM1, displaced gigantic M1. Scale bars: A, 100 μm; B, 80 μm; C, 50 μm [Figure adapted from Hannibal et al. (23); courtesy of Dr. J. Hannibal and Journal of Comparative Neurology].

The morphological characterization of ipRGCs in the human retina has now advanced substantially; several recent studies provided a detailed morphological description of ipRGCs in the retina of human donors (Figure 1) (22–25, 37). In humans, the reported number of ipRGCs varies from ~4,000 to more than 7,000, but it remains extremely marginal (0.4–1.5%) compared to the 1.07 million ganglion cells in the human retina (22–24, 35, 38, 39). Two distinct morphological types roughly correspond to the M1 type of the mice, with dendrites that are primarily or exclusively in the outer sublamina of the inner plexiform layer (IPL), and the M2 type of the mice with dendrites that are primarily or exclusively in the inner sublamina of the IPL (40). The fovea is devoid of ipRGCs. The ipRGCs are most abundant in the peri-foveal region (~15–40 cells/mm^2^) and their number declines to <5 cells/mm^2^ at 10 mm eccentricity and beyond (23–25); in that, they parallel the decrease of density of RGCs from the center to periphery of the retina. Additional morphological subtypes of ipRGCs have been reported in specific studies including M3, M4, and types that further subdivide M1 type into standard M1, gigantic M1, displaced M1 (dM1), and gigantic dM1 (22–25) (Figure 2). Of note, in human, but not in the mouse, dM1 constitute the majority of M1. Importantly, these morphological studies relied on immunostaining of melanopsin, a method that, in mice, has been shown to fail to detect all ipRGCs [see Aranda and Schmidt (19)]. This suggests a probable underestimation of the total number of ipRGCs and potential bias in the reported subtype distribution.

## ipRGCs Brain Targets

Mapping the projections of ipRGCs in the brain has been instrumental to discover their multiple functions. In the mouse, ipRGCs convey light information to more than a dozen brain regions, including several nuclei implicated in circadian rhythms [suprachiasmatic nucleus (SCN), intergeniculate leaflet (IGL)], sleep and wake regulation [in the hypothalamus, the ventrolateral preoptic area (VLPO) and lateral hypothalamus (LH), and the centro-medial nucleus in the thalamus], PLR control [olivary pretectal nucleus (OPN)], and mood (peri Habenula) (41–44). Visual structures such as the dorsal lateral geniculate nucleus (dLGN) and the superior colliculus (SC) are also targeted.

In human, the exploration of ipRGC projections is limited by the impossibility to use the appropriate techniques, e.g., injection of tracers or genetically encoded labels. However, Hannibal and colleagues (35) took advantage of the fact that the pituitary adenylate-cyclase-activating polypeptide (PACAP) is a marker for retinohypothalamic tract (RHT) projections to the SCN in rodents and human (45) and that PACAP is found in virtually all ipRGCs in the retina of human to describe ipRGC putative projections on the SCN. They found a dense terminal field of PACAP-positive nerve fibers in the retinorecipient zone (ventral part) of the SCN in two human donors (while no PACAP-immunoreactive cell bodies were found in the SCN). The fibers mainly arose from the optic chiasma and were found in close apposition to VIP-containing neurons in the ventral SCN.

Given the impossibility to use tracers in humans, studies in non-human primates remain essential for completing the mapping of ipRGC central projections in the primate. Classical retrograde tracing from the lateral geniculate complex and the pretectum in macaque identified these areas as targets for the ipRGCs (34). Using immunohistochemical staining of PACAP in combination with staining for the anterograde tracer (Cholera Toxin Fragment B) delivered by intraocular injection, ipRGC projections to the SCN were confirmed in macaque (46). Additionally, projections to the LGN including the pregeniculate nucleus [which is thought to correspond to the rodents IGL (47)], the OPN, the nucleus of the optic tract, the brachium of the SC, and the SC were identified (46). Interestingly, in the macaque, ipRGC projections to the dLGN emerge from both inner and outer stratifying melanopsin cells (hence potentially from all ipRGC subtypes), while in the mouse, the majority of melanopsin ganglion cell innervation of the dLGN appears to be provided only by inner stratifying cells [non-M1 cells (41, 44, 48)]. Whether this discrepancy reflects an extended role of ipRGCs in vision in the primate remains to be clarified. Finally, in the mouse, ipRGC terminals are found in numerous hypothalamic nuclei in addition to the SCN, including the VLPO, LH, anterior hypothalamic nucleus, ventral subparaventricular zone, and peri-supraoptic nucleus (42, 44). Retinal projections to these hypothalamic nuclei also exist in the primate (49, 50). However, whether these projections include ipRGCs remains to be verified. It is not a trivial question as these nuclei often heavily influence physiology through the control they exert on sleep, appetite, and thermoregulation to name a few.

## Functional Properties and Diversity of Human ipRGCs

The first report of human RGCs direct electrophysiological recording was published by Weinstein et al. (51). This study measured the spectral sensitivity of two RGCs around the photopic peak (555 nm). However, such recordings in the human retina would then remain anecdotal until recently. There have been as many studies, peer-reviewed articles and non-peer-reviewed, preprint manuscripts, on the human retina physiology over the last 2 years as in the previous 50 years (52–57).

So far, only one study has been specifically designed to capture human RGCs' intrinsic sensitivity and to describe ipRGC responses to light and functional diversity (55). Overall, the characteristic features of pharmacologically isolated human ipRGC responses, i.e., when their response is solely driven by melanopsin, seem similar to that of rodents and macaque (17, 34, 58, 59). Human ipRGCs' intrinsic responses to light are slow, sustained over the entire stimulation, and do not extinguish immediately after light OFF. These kinetic properties make ipRGC responses very different from rod- and cone-driven responses that are extremely fast (<100 ms). Intrinsic photoresponses of human ipRGCs are reversibly inhibited by opsinamide, a drug that specifically blocks melanopsin (60). Mure et al. also found that ipRGCs' intrinsic sensitivity was low; ipRGCs did not seem to respond to light intensities below photopic level, even following dark adaptation. Their spectral sensitivity peaked in the blue region of the spectrum (~460 nm), different from the peaks of human rods and cones but close to mouse and macaque melanopsin peaks (17, 34) and to the human melanopsin expressed in HEK293 cells (61). This result is also consistent with ipRGCs' role in human non-visual responses to light such as nocturnal melatonin peak suppression (62, 63), PLR (64, 65), non-cone/non-rod visual awareness (13, 66), cognition (67), and heart rate modulation (68) that are also maximally sensitive to blue light.

**Figure 3:**
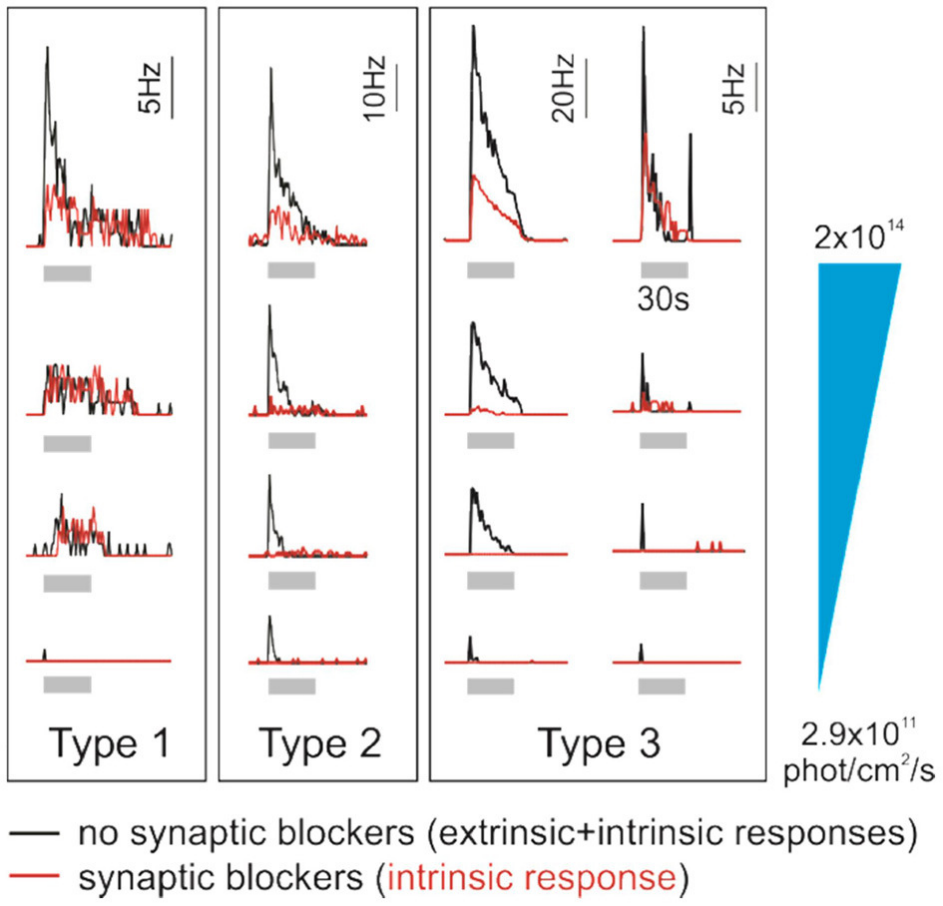
Human ipRGCs integrate extrinsic signals. Individual examples of type 1, 2, and 3 ipRGCs' responses to increasing irradiance light pulses (gray bars, 30 s, 470 nm; from bottom to top, irradiance is 2.9 × 10^11^, 3.5 × 10^12^, 2 × 10^13^, and 2 × 10^14^ photons/cm^2^ per second). Red traces represent the responses of pharmacologically isolated ipRGCs, which reflect their intrinsic photosensitivity. In contrast, black traces report the responses from the same cells in the absence of synaptic blockers and thus integrating input from outer retina photoreceptors. Time course, sensitivity, and intrinsic properties of the response differ between the ipRGC subtypes. The contribution from rods/cones to the overall ipRGC responses to light also seems to be subtype-specific. Interestingly, ipRGC subtypes may receive different inputs from photoreceptors. Of note, in human, morphological and functional ipRGC subtypes are not yet fully consolidated; here, ipRGC subtypes are labeled as in the original study from which this figure is adapted (55).

Human ipRGCs' response parameters and time courses suggest that they consist of several functional groups. Mure et al. described three ipRGC subtypes, each one displaying unique response kinetics and sensitivity to light (Figure 3). Type 1 ipRGCs are more sensitive to light and sustain response long after the light is turned off. Type 2 ipRGCs are less sensitive and turn OFF faster. At low irradiance levels, type 2 ipRGCs exhibit longer response latency to the test light pulse. Type 1 responses are recorded 50% more frequently than Type 2 responses. A third type of ipRGCs responded only in the presence of exogenous chromophore (11-cis retinal) in the medium. These Type 3 cells responded more strongly, but only to the high irradiance levels, and extinguished faster after light OFF. Altogether, the features of Type 1, Type 2, and Type 3 ipRGCs suggest that they could correspond to mouse ipRGC subtypes that have been labeled M1, M2, and M4 ipRGCs, respectively (69–71). However, the link between the human physiological and morphological ipRGC subtypes, and their correspondence with the murine subtypes, remains to be established. Also, Mure et al.'s study was performed on a limited number of donors; these findings need to be independently replicated. The effort must be pursued to refine the results and to increase the number and diversity of donors. Recently, light-induced melatonin suppression in the evening, a process under ipRGCs control, has been shown to vary up to 50 times between subjects (72). It would be interesting to determine to which extent ipRGCs contribute to such variability in light sensitivity (73).

## Transcriptome Diversity of Human ipRGCs

Underlying the morphological and functional diversity are the different gene expression profiles of ipRGCs. In mice, the first indication of the molecular heterogeneity of ipRGCs came with the observation that all ipRGCs express the transcription factor Brn3b except for the fraction of M1 cells that project to the SCN (74). Thus, while all M1 ipRGCs are morphologically and electrophysiologically similar, two molecularly different subpopulations co-exist and innervate different brain regions (SCN for M1 Brn3b– and OPN for M1 Brn3b+). This additional dimension of identity is now easily approachable. High-throughput methods [single-cell RNA sequencing (scRNAseq) or RNAseq applied on RGCs-enriched samples] allowed distinguishing several ipRGC subpopulations in both mouse and primate (75–79).

In macaque and human retina, scRNAseq performed on CD90+ cells to enrich the samples with RGCs (CD90 or Thy1 is a cell surface protein marker of RGC class) allowed differentiating up to 18 RGCs subpopulations (77, 78). The four most abundant RGC clusters were easily identified as ON and OFF midget RGCs and ON and OFF parasol RGCs that account for respectively >80% and ~10% of all RGCs in the primate retina. The remaining RGC clusters each consists of ~1% or less of all RGCs. Melanopsin was expressed at detectable levels in a few of these RGC clusters in the peripheral retina, three in the macaque (77) and two in human (78). In human, the authors noted a sensible difference in expression levels of melanopsin and hypothesized a correspondence between the cluster expressing the highest level of melanopsin and M1 ipRGCs, which express the highest levels of melanopsin in mice (20), while other subtypes (M2–M6) would constitute the remaining cluster or be too rare to be detected.

Interestingly, the comparative study of murine and macaque retina cell transcriptomes indicates that the ganglion cells are the less conserved retinal cell type between the two species. However, while conventional RGCs only show weak correspondence in terms of both diversity and distribution, ipRGCs seem to be among the most conserved features (77, 79). This may reflect the differences in the visual signal tracked by nocturnal and diurnal animals and thus in the organization of their respective visual systems. In contrast, the features of the light signal relevant to non-visual responses such as the ambient level of light for the circadian system are similar for most organisms and may rely on similar cell types.

## Integration of External Input From Photoreceptors

In a similar way to conventional RGCs, ipRGCs convey rod- and cone-initiated photoresponses and integrate these extrinsic signals and their intrinsic photosensitivity (80, 81). The contribution of outer retina photoreceptors to human ipRGC signaling can be studied by comparing ipRGC responses before and after application of synaptic blockers that isolate RGCs from extrinsic input (55) (Figure 3). It is important to keep in mind, however, that the photoreceptor responses may be differentially affected by the preparation itself. For example, in the absence of RPE *in vitro*, the input from rods and cones may be diminished and their contribution may be underestimated. In the absence of synaptic blockers, a large number of RGCs respond to light. Most of them become silent after incubation with blockers as conventional RGCs do not receive rod and cone signals anymore. ipRGC responses persist; however, their response is generally altered. More specifically, the response threshold is higher and the latency is longer while the amplitude is decreased. Of note, the part of rod and cone responses in the overall response seems to be specific to the ipRGC subtypes. For all subtypes, extrinsic input to ipRGCs shortens the response latencies and lowers the response thresholds. However, only for Type 2 and 3 ipRGCs did the extrinsic input account for a significant portion of the sustained response and increase their sensitivity. A similar observation was made in the mouse where the contribution of rods and cones to ipRGC responses seems inversely proportional to melanopsin photosensitivity; while mouse M1 ipRGC responses are moderately influenced, the M2–M5 subtype responses rely more heavily on extrinsic inputs (82). The response of Type 3 ipRGCs, in particular, seems to rely the most on input from rods/cones, which is in line with the description of M4 ipRGCs (83, 84). Type 1 ipRGCs receive only minimal extrinsic inputs compared to other subtypes. M1 ipRGCs, which may be the mouse orthologous of human type 1 ipRGCs, are sufficient to photoentrain the clock (74). This is consistent with the finding that cones, while they may contribute to the entrainment of the clock in humans (85), are not required for it (86). As mentioned above, human and mouse cones differ in number and peak wavelength sensitivity, which suggests different weights of their input to ipRGCs in response to the same light stimulus. There may also be important functional divergences. For example, short-wavelength cones and melanopsin are antagonistic in controlling the primate PLR but additive in the murine PLR (87, 88). This illustrates the importance of elucidating the subtype-specific contribution of rods and cones as they can dramatically alter ipRGC spectral sensitivity; i.e., they can shift their action spectra from blue toward shorter or longer wavelengths.

Overall, the rod/cone input to ipRGCs expands the dynamic range of irradiance and temporal frequencies over which the ipRGCs signal (17, 34, 55). The diversity in ipRGC subtypes combined with the way they specifically integrate rod and cone signals could explain their ability to regulate such a variety of responses to light functioning at various time constants and light levels.

## ipRGCs in Aging and Disease

Several recent studies have highlighted the progressive loss of ipRGCs with aging, which is aggravated in neurodegenerative diseases (22, 89–92). A decrease in the total number of ipRGCs and the size of dendritic arborization occurs progressively with aging [31% loss in healthy subjects older than 70 years (22)]. However, there are conflicting reports about the functional significance of such decline. Some reports suggest that ipRGC response properties might show a functional compensation by increasing their sensitivity and/or firing rate so that no significant change in ipRGC-dependent response such as PLR is observed in older individuals (93, 94). However, there are also reports of reduced amplitude of circadian rhythm in body temperature and increasing prevalence of sleep fragmentation among the elderly (95, 96), which can be improved by bright light (8). ipRGC responses measured directly in an old donor (>70 years) display longer latency (i.e., it responds slower to a light pulse) and overall shorter duration (55). While this observation needs to be confirmed, it suggests that not only ipRGCs' number but also their function may be altered in aging.

The specific loss of ipRGCs observed with aging is accelerated in Alzheimer's and Parkinson's diseases (AD and PD). AD and PD patients have 25–30% fewer ipRGCs compared to healthy age-matched controls (37, 90), and surviving ipRGCs display dendritic processes. Protein aggregates have been observed in and around ipRGCs of AD patients and may be the cause of altered neuronal physiology (97). These results suggest that ipRGC degeneration may lead to circadian rhythm and sleep dysfunction in neurodegenerative disorders (89, 98). In glaucoma, ipRGCs, while initially more resilient than conventional RGCs, are lost at advanced stages (91). Finally, a dramatic loss of ipRGCs is observed in diabetic retinopathy; however, it correlates with the overall loss of RGCs (92). In summary, histological assessments show a decline in the number of ipRGCs in old age and neurodegenerative diseases. Although some evidence suggests that ipRGCs' function is also altered in old age, whether the ipRGCs' intrinsic light response, the input of rod and cones, and/or the abundancy of ipRGCs subtypes are affected during aging and neurodegeneration remains to be investigated.

Of note, ipRGCs are not always more vulnerable than conventional RGCs; they possess a higher ability to survive certain pathological and experimental conditions. In the mouse, ipRGCs appear more resistant than other RGCs to various insults, including optic nerve injury, glutamate-induced excitotoxicity, and early-stage glaucoma (99, 100). In human patients, ipRGCs resist neurodegeneration in two inherited mitochondrial disorders that cause blindness: Leber hereditary optic neuropathy and dominant optic atrophy (101). This ability seems to be independent from melanopsin expression *per se* as ipRGCs' resilience is preserved in a mouse model bearing the mutation causing dominant optic atrophy and lacking melanopsin (102). Specific metabolic properties, such as higher mitochondrial activity or content, have been hypothesized as potential neuroprotective mechanisms. However, the reason why ipRGCs are relatively spared is still not well=understood.

The peculiar behavior of ipRGCs (i.e., increased vulnerability or resilience to certain disorders) compared to conventional RGCs has important implications. First, a better molecular characterization of each ipRGC subtype across aging and diseases will allow identifying the expression programs associated with differential cell survival and will provide therapeutic targets to diminish the loss of vision following optic nerve injury or ocular disease (100). Then, ipRGCs could be a promising marker to assess CNS disorders, corroborating the old saying that the eyes are a window to the soul (103, 104). The idea is appealing when one considers that PLR is a cost-efficient, fast, non-invasive readout of ipRGCs' function (64, 65). The PLR assay is now considered an emerging method to assess retinal and CNS disorders (105, 106) and has been suggested in the context of neurodegeneration as potential diagnostic or follow-up tools (107, 108). This translation has been unsuccessful with AD so far (109, 110), but this may just emphasize the need for direct measurements of ipRGCs' function in patient donors. These data would allow precisely pointing out the part of the response that is altered and designing more suited stimulation protocols that target it. A limitation might be that PLR relies on, and consequently will inform only on, specific ipRGC subtypes (part of M1 and M2 ipRGCs); it cannot be generalized as a proxy for all ipRGCs and thus will not be predictive of all ipRGC-dependent disorders.

## Concluding Remarks

Knowledge of human ipRGCs is now catching up with what we know of these cells in the mouse. To date, these results emerge from a still limited number of labs; they would need to be replicated. Some points also remain to be clarified; for example, regarding the existing ipRGC's populations. Does the M3 subtype detected in some studies constitute a real ipRGC's subpopulation in human (22) or are the few resembling cells just marginal between M1 and M2 (24)? M4 are only described by one group (23) while M5 and M6 ipRGCs have not been described yet in the human retina. Does it mean that these ipRGC subtypes do not exist, are not morphologically distinct or too rare, and may be discovered later as in the mouse? Then, how do the projection maps compare? ipRGCs seem to target the same visual structures in both mouse and human while the subtypes of cells are not necessarily the same. Whether the numerous hypothalamic projections observed in the mouse translate in human (other than the SCN) need to be confirmed. This is particularly important given the control exerted by the hypothalamus over the body homeostasis and behaviors. Finally, a challenge that applies not only to human ipRGCs but also to the field, in general, is to consolidate ipRGC subtype classification by reconciling morphological, functional, and transcriptional identities. New approaches like patch-seq that combines scRNA-seq profiling with electrophysiological and morphological characterization of individual neurons may be an approach to consider (111, 112). This would constitute the first step toward completing the assignment of a specific function (and potential role in disorders) to each ipRGC subtype and fully elucidating both the circuits up- and downstream of each ipRGC subtype.

The differences that emerged between mouse and primate highlight the compelling need to include human donor retina in the standard models. Non-human primates remain necessary for some studies like mapping the projections. However, they are not advantageous ethically or economically over human preparations and consequently do not allow for a larger sample size. Furthermore, the tissue collection can be planned and operated within similar delays in monkey and human, at least for the surgical samples. The parameters affecting the fitness of the preparation may thus be controlled (hypoxia delay, pH, or nutrients) (52, 53). Human ipRGC exploration may also include the development of additional human *ex vivo* and *in vitro* models such as long-term culture of retina or retina organoids. Some results are very encouraging as retina organoids are photosensitive, organized in layers, and display a cellular diversity that partly recapitulates the diversity of functional peripheral retina (53).

There is a strong incentive to pursue these efforts as this handful of cells plays a major role in our physiology, cognitive performances, and overall well-being. Also, as progress in lighting science now allows for precise manipulation of quality, quantity, and timing of light, understanding how ipRGCs operate in the human eye in health and diseases will enable new applications. For example, the insights could be used to design indoor lights that offer better day–night synchronization or which improve our moods. It will offer a framework for improving the “spectral diet” of human at home, at work, or in public spaces (113).

## Author Contributions

The author confirms being the sole contributor of this work and has approved it for publication.

## Conflict of Interest

The author declares that the research was conducted in the absence of any commercial or financial relationships that could be construed as a potential conflict of interest.
